# 肺癌术后支气管胸膜瘘致死性大咯血经验（附7例报告）

**DOI:** 10.3779/j.issn.1009-3419.2012.01.08

**Published:** 2012-01-20

**Authors:** 真铭 张, 允 王

**Affiliations:** 610041 成都，四川大学华西医院胸外科 Department of Thoracic Surgery, West China Hospital of Sichuan University, Chengdu 610041, China

**Keywords:** 肺肿瘤, 支气管胸膜瘘, 支气管血管瘘, 咯血, Lung neoplasms, Bronchopleural fistula, Bronchovascular fistula, Hemoptysis

## Abstract

**背景与目的:**

大咯血是肺癌术后少见但严重的并发症。本研究旨在探讨致死性大咯血的发生机制、危险因素、先兆症状及预防和治疗措施。

**方法:**

2007年4月-2011年5月四川大学华西医院共行肺癌手术1, 737例，围手术期死亡20例，其中死于大咯血7例，复习7例患者的临床资料并结合文献进行分析。

**结果:**

大咯血是肺癌术后第2位死亡原因。7例中6例直接死于大咯血，1例因大咯血行二次手术，最终死于肺部感染、呼吸衰竭。4例发生过先兆出血症状。4年大咯血发生率为0.4%（7/1, 737）。

**结论:**

支气管胸膜瘘引起的支气管血管瘘是大咯血发生的机制，糖尿病为高危因素，早期诊断、早期外科治疗支气管胸膜瘘或支气管血管瘘可避免大咯血死亡的发生。

## Abstract (en)

**Background and objective:**

Massive hemoptysis was a rare but severe postoperative complication of lung cancer. The aim of the present study is to investigate the mechanisms, risk factors, early symptoms, prevention, and treatment options for fatal hemoptysis.

**Methods:**

From April 2007 to May 2011, 1, 737 patients with lung cancer were surgically treated in the West China Hospital of Sichuan University. Twenty patients died during the perioperative period, seven of whom died of massive hemoptysis. These seven cases were analyzed, and their clinical data, as well as related literatures, were reviewed.

**Results:**

Massive hemoptysis is the second cause of death after lung cancer surgery. Six patients died directly of massive hemoptysis. One patient underwent secondary surgery because of massive hemoptysis, but eventually died because of lung infection and respiratory failure. Early symptoms of hemorrhage were observed in four cases, and the overall incidence rate of massive hemoptysis was 0.4% (7/1, 737).

**Conclusion:**

Bronchovascular fistula (BVF) caused by bronchopleural fistula (BPF) is the mechanism for massive hemoptysis. Diabetes is a high risk factor. Early diagnosis and surgical treatment of BPF or BVF can prevent the occurrence of death as a result of massive hemoptysis.

支气管血管瘘（bronchovascular ﬁstula, BVF）是肺癌术后一种少见但极其严重的并发症，可导致大咯血的发生，且通常是致命的^[1-3]^。2007年4月-2011年5月四川大学华西医院胸外科共行肺癌手术1, 737例，其中支气管袖式肺叶切除87例，术后发生大咯血6例，肺叶切除1540例，术后发生大咯血1例。我们回顾性分析该7例患者的临床资料并结合文献复习，旨在探讨肺癌术后大咯血发生的机制、危险因素、先兆症状及预防和治疗措施，供临床借鉴。

## 1 资料与方法

回顾性分析2007年4月-2011年5月在四川大学华西医院胸外科治疗的20例围手术期死亡肺癌病例，均为男性，其中死于肺部感染、呼吸衰竭11例，死于大咯血7例，死于多器官功能衰竭1例，死于左心衰竭1例。13例非咯血死亡患者无1例有糖尿病病史，7例咯血死亡患者中6例有糖尿病病史。7例咯血死亡患者年龄47岁-61岁，平均年龄55岁。7例中行支气管袖式肺叶切除、淋巴结清扫术6例，其中右肺手术4例，左肺手术2例；行胸腔镜（video-assisted thoracic surgery, VATS）左肺下叶切除、淋巴结清扫术1例。7例肺癌手术由2个医疗组完成，袖式肺叶切除的支气管吻合口均采用3/0 prolene线连续缝合。支气管吻合口使用可吸收聚乙醇酸修补材料（absorbable polyglycolic acid felt, APAF）包裹处理4例，使用心包处理1例，使用奇静脉处理1例，VATS肺叶切除患者的支气管残端未行包裹处理（表 1）。

**1:** 7例咯血患者的临床资料 Clinical data of the seven patients

Number	Sex	Age	Diabetes	Operation time	Operation	Stump or anastomosis coverage	Early symptoms of hemorrhage	BPF（Fiberoptic bronchoscopy demonstrated）	Empyema	Reoperation	Death time	Cause of death
1	Man	59	Yes	February 21, 2011	Right upper sleeve lobectomy	APAF	No	Yes	No	No	March 4, 2011	Fatal hemoptysis
2	Man	60	Yes	July 26, 2010	VATS left lower lobectomy	No	No	Yes	No	No	August 11, 2010	Fatal hemoptysis
3	Man	61	Yes	April 9, 2007	Left upper sleeve lobectomy	APAF	No	No	Yes	No	May 3, 2007	Fatal hemoptysis
4	Man	47	Yes	March 11, 2011	Right upper sleeve lobectomy	Azygos vein	Yes	Yes	Yes	No	April 9, 2011	Fatal hemoptysis
5	Man	52	Yes	May 11, 2011	Right upper sleeve lobectomy	APAF	Yes	No	Yes	Yes	June 9, 2011	Pneumonia and respiratory failure
6	Man	57	Yes	March 1, 2011	Left upper sleeve lobectomy	Pericardium	Yes	No	No	No	April 13, 2011	Fatal hemoptysis
7	Man	49	No	October 17, 2007	Right upper sleeve lobectomy	APAF	Yes	No	No	No	November 18, 2007	Fatal hemoptysis
VATS: video-assisted thoracic surgery; APAF: absorbable polyglycolic acid felt.												

## 2 结果

### 2.1 临床表现

7例患者发生大咯血前的临床症状：发热6例；明显呼吸困难4例；刺激性咳嗽2例；先兆出血4例（表 1），包括痰中陈旧性血丝1例，先兆咯血3例，手术切口大量渗鲜血2例，引流管引流出鲜血1例；引流管引流出脓性积液1例；手术切口渗出脓性积液1例。

### 2.2 支气管镜检查

7例患者术后行纤维支气管镜检查6例，其中由纤维支气管镜证实的支气管胸膜瘘（bronchopleural fistula, BPF）3例，包括袖式肺叶切除2例，VATS肺叶切除1例（表 1）。纤维支气管镜检查发现支气管吻合口缺血坏死2例，其中1例袖式肺叶切除患者的连续纤维支气管镜检查观察到了支气管吻合口逐渐缺血坏死的过程（图 1）；残端支气管完全坏死1例（图 2）。早期纤维支气管镜检查未有明显异常2例。

**1:**
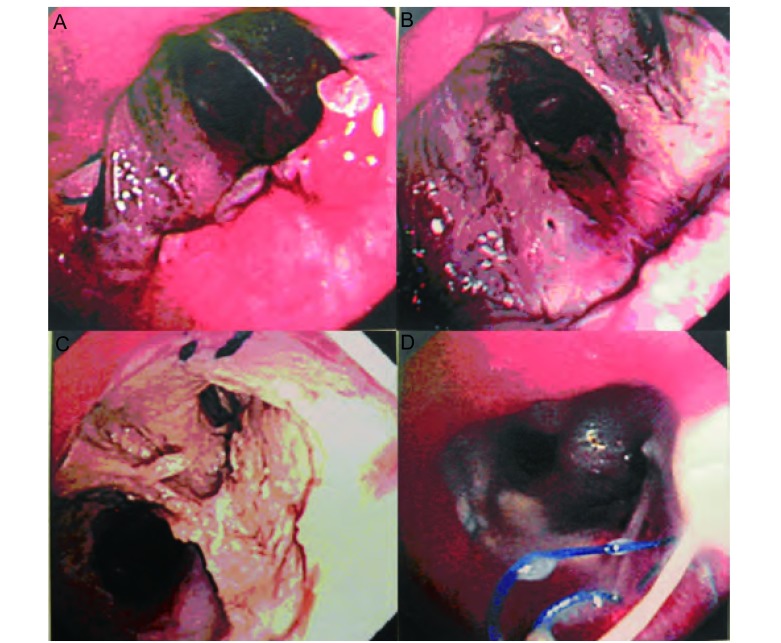
袖式肺叶切除术后的连续纤维支气管镜检查（男，47岁）。A：支气管吻合口闭合良好，中下叶支气管粘膜光滑（术后4天）；B：右中下叶支气管粘膜部分坏死（术后7天）；C：右肺残断支气管粘膜完全坏死（术后17天）；D：支气管胸膜瘘形成（术后26天）。 Sequential fiberoptic bronchoscopy after sleeve lobectomy (male, 47 years old). A: Well close of the bronchial anastomosis and smooth middle and lower lobe bronchial mucosa (4^th^ postoperative day); B: Partial mucosal necrosis of the right middle and lower lobe bronchus (7^th^ postoperative day); C: Complete mucosal necrosis of the right residual bronchus (17^th^ postoperative day); D: Formation of the bronchopleural fistula (26^th^ postoperative day).

**2:**
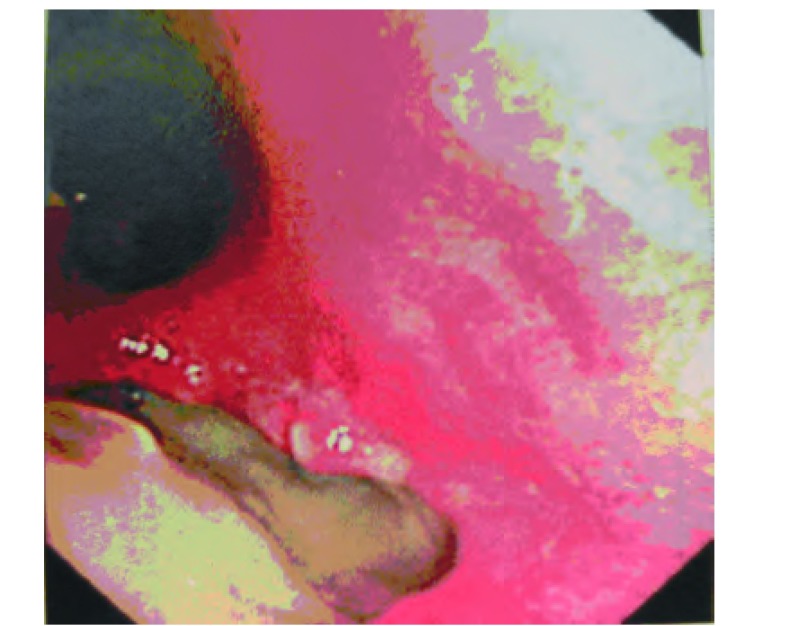
VATS肺叶切除术后支气管胸膜瘘（男，60岁）。纤维支气管镜示左下叶支气管胸膜瘘，残端支气管完全坏死（术后10天）。 Bronchopleural fistula after VATS lobectomy (male, 60 years old). Fiberoptic bronchoscopy shows bronchopleural fistula of the left lower lobe bronchus and complete necrosis of the residual bronchus(10^th^ postoperative day).

### 2.3 结局

7例咯血患者均死亡，包括直接死于大咯血6例，死于二次术后的肺部感染、呼吸衰竭1例（术后发生BPF）。死亡时间分别是术后第11、16、24、29、29、30、32天，最早11天，最晚32天，平均24.4天（表 1）。

## 3 讨论

肺癌术后大咯血是一种少见的并发症，可致患者突然死亡，后果严重^[1-3]^。袖式肺叶切除术后的支气管吻合口靠近肺动脉，故大咯血一般发生在支气管袖式肺叶切除患者，被认为是支气管袖式肺叶切除的缺陷^[2]^。本组支气管袖式肺叶切除术后总的4年大咯血发生率为6.90%（6/87）。目前尚无文献报道肺叶切除术后的大咯血，本组7例咯血患者中仅有1例为肺叶切除患者，肺叶切除术后总的4年大咯血发生率为0.06%（1/1, 540），明显低于袖式肺叶切除，这是手术方式的不同决定的。

本组7例患者中由纤维支气管镜证实的BPF 3例，1例因先兆咯血行二次手术，术中发现支气管胸膜瘘与支气管动脉瘘形成，吻合口旁包裹性积脓，肺动脉被腐蚀，有1例虽无直接的BPF证据，但有脓胸的证据，另外2例有BPF的临床表现，而同期的13例非咯血死亡病例无1例发生BPF。因此，考虑是BPF引起的支气管周围脓肿腐蚀肺动脉形成BVF，导致大咯血的发生。BVF典型的症状为突发气道大出血，通常是致命的大咯血，本组7例咯血患者中有6例直接死于大咯血，是本科室肺癌术后第2位死亡原因（7/20）。

肺叶切除术后BPF的发生与很多因素有关，缺血是BPF发生的重要原因之一^[4, 5]^。缺血通常表现为支气管管壁出现溃疡或者坏死^[6]^。本组病例中VATS肺叶切除术后10天的纤维支气管镜发现了完全坏死的残端支气管。因此认为缺血是此病例发生BPF的主要原因。对于支气管袖式肺叶切除，发生吻合口BPF的主要原因也是因为缺血^[1, 3]^。本组病例中6例行袖式肺叶切除术，其中1例患者的4次连续纤维支气管镜检查观察到了支气管吻合口逐渐缺血坏死的过程，另1例袖式肺叶切除术后6天的纤维支气管镜检查同样发现了支气管吻合口愈合不良、远端坏死的证据，证实了缺血是支气管袖式肺叶切除发生BPF的主要原因。

糖尿病是肺部手术发生BPF的危险因素之一，可致支气管愈合不良，并增加BPF的发生风险^[6-8]^。本组7例病例中6例有糖尿病病史，而13例非咯血死亡病例均无糖尿病病史，也无1例发生BPF。因此糖尿病可能促进了本研究中BPF的发生，从而导致了BVF的发生，是BVF发生的危险因素。

显然，要预防BVF的发生，关键是要预防BPF的发生。缺血是本组病例发生BPF的主要原因，文献^[1, 3]^报道缺血的发生是过度破坏支气管动脉的后果，这常发生于过度分离支气管表面组织及广泛的淋巴结清扫时。因此，手术分离支气管时不能把支气管过度骨骼化而造成支气管表面滋养血管的破坏，在清扫淋巴结时也要注意保护支气管动脉不被广泛破坏。本研究中支气管袖式肺叶切除术后BVF的发生率为6.90%（6/87），高于文献^[9]^报道的2.0%，这可能与支气管动脉的过度破坏有关。支气管动脉起自主动脉、肋间动脉，沿支气管进入肺门，延伸于支气管表面形成血管网，并且支气管动脉的分支与肺动脉的分支尚存在吻合，形成支气管肺循环^[10]^。有研究^[11]^表明在阻断支气管动脉后因支气管肺循环逆流血运的产生而不会造成支气管缺血改变。因此，我们认为只要不过度破坏吻合口两断端支气管表面的滋养血管就不会造成吻合口的缺血。

本组6例袖式肺叶切除由2个医疗组完成，6例支气管吻合均使用3/0 prolene线连续缝合。支气管吻合的连续缝合技术早已被证实是一种安全的吻合技术，且有简单、快速、经济的优点，可以取得与间断缝合相同的手术结果^[12]^。华西医院胸外科自2008年以来一直采用连续缝合的方法，取得了令人满意的手术结果，因此认为连续缝合不是本组吻合口缺血及BPF发生的直接原因。

另外，避免在张力下吻合支气管是防止吻合口并发症的另一重要措施。吻合口张力过大常是过度切除支气管的结果，张力过大可导致支气管吻合口裂开^[1]^，从而继发BPF和BVF^[9]^。手术时既不能过度切除支气管以保证断端无癌细胞残留，也不能因切除长度不够而致断端癌细胞残留。总之，防止吻合口并发症的最重要措施是保护支气管血管并避免在张力下做支气管吻合^[9]^。

对于高危发生BPF的患者（如糖尿病患者），带蒂肌瓣应该常规用于手术^[13]^。对于支气管袖式肺叶切除，带蒂肌瓣包裹吻合口，使得吻合口和肺动脉相隔开，这样既可以避免BPF的发生，也能防止BVF的发生。另有研究^[3, 9]^表明防止BVF的最重要措施是用血供丰富的组织包裹支气管吻合口，这样既可促进支气管愈合，又可在吻合口和肺动脉之间形成一个屏障。本组VATS肺叶切除病例的支气管残端未行包裹处理，这与腔镜下操作的局限性有关。6例袖式肺叶切除中有4例使用APAF包裹支气管吻合口，可见，APAF因不含血运而并不能有效保护吻合口。另外2例中1例使用心包，有研究^[13]^认为心包也并不能提供足够的保护，血供也不够丰富；1例使用奇静脉，奇静脉的缺点在于只能用于右肺手术，且面积也不足够大，并不比带蒂肌瓣更好。带蒂肌瓣中，前锯肌、背阔肌及肋间肌都可供选择，一方面足够大，可以顺利到达胸腔，另一方面富含血运^[14]^。带蒂肌瓣选择方面，Rea等^[9]^报道对高危患者常规使用肋间肌后，就不曾发生过BVF。Cerfolio等^[13]^也倾向选择肋间肌，认为肋间肌既可靠又方便移植。

BPF发生时可出现一些临床症状，如发热、呼吸困难、刺激性咳嗽、痰中陈旧性血丝、引流管引流出脓性积液等^[8, 14]^。当怀疑有BPF发生时，应该立即行支气管镜检查，而且应该越早越好^[14]^。BVF引起的致命大咯血发生前也可出现一些先兆出血症状，如先兆咯血（咯血量一般不大，不至于使患者立刻死亡）、引流管引流出鲜血、切口渗鲜血等。同样，当患者术后发生先兆出血症状时，也要怀疑BVF发生的可能，应立刻行支气管镜检查以明确诊断^[15]^。

本组死亡病例证实了一旦BPF或BVF诊断成立，及早的外科介入是非常必要的。对于BPF的成功治疗基于早期的诊断、充分的胸腔引流、适当的抗菌药物控制感染、及早再次手术关闭瘘口（患者的全身情况允许，胸腔引流充分，感染得到控制）以及带蒂肌瓣的运用及残腔的消灭^[8, 14]^。对于肺叶切除患者，早期的手术、残端重新关闭最早可以在术后7天-14天完成，重新关闭的残端也应该用带蒂肌瓣包裹，没有带蒂肌瓣覆盖不可避免会再次发生BPF^[14]^。如前所述，前锯肌、背阔肌及肋间肌都可供选择，并且带蒂肌瓣还可用于术中填塞消灭残腔。本组VATS肺叶切除病例术后10天的纤维支气管镜检查证实了BPF的发生，但未采取及时的外科手术治疗，最终导致咯血死亡。对于袖式肺叶切除患者，完全坏死的气管、支气管不允许再次吻合，全肺的切除可能不可避免，同样，支气管残端也应该用带蒂肌瓣包裹，残腔可用肌肉填塞，适当的胸廓成型术也可用于消灭剩余的残腔^[14]^。本组袖式肺叶切除病例中，5例未采取及早的手术治疗，最终也导致了咯血死亡。有1例虽行了二次手术（右残肺及隆突切除、气管左主支气管吻合术），但术后再次发生BPF（气管插管内大量血性胸腔积液），最终死于肺部感染、呼吸衰竭。该例患者二次手术时的吻合口未使用肌瓣包裹可能是发生BPF的重要原因之一。

总之，BVF是肺癌术后一种少见但极其严重的并发症，多数发生于袖式肺叶切除，被认为是袖式肺叶切除术的缺陷^[2]^，表现为致命大咯血。BPF引起的BVF是大咯血发生的机制，只有预防BPF的发生才能预防BVF的发生。缺血是本组病例发生BPF的主要原因，故手术时应该小心保护支气管动脉不被广泛破坏。糖尿病作为危险因素可增加BPF的发生风险，是BVF发生的危险因素，对于高危患者，带蒂肌瓣应该常规用于手术。当怀疑有BPF或出现大咯血的先兆出血症状时，应该立即行支气管镜检查，早期诊断、早期外科治疗BPF或BVF可避免大咯血死亡的发生。
